# The relationship between maternal and infant empathy: The mediating role of responsive parenting

**DOI:** 10.3389/fpsyg.2022.1061551

**Published:** 2022-12-22

**Authors:** Hang Liu, Yuning Zhu, Xiaoqi Cai, Zhengmei Ma, Lu Wang

**Affiliations:** ^1^Faculty of Education, Northeast Normal University, Changchun, China; ^2^School of Psychology, Northeast Normal University, Changchun, China

**Keywords:** maternal empathy, infant empathy, responsive parenting, situational observation, parent–child interaction

## Abstract

Individual empathy emerges during infancy, and its development is influenced by family environmental factors such as parental characteristics and parenting style. In this study, we examined how maternal empathy was related to infant empathy and the mediating role of responsive parenting in this relationship using situational observation and scale measurement data. Thirty-three infants aged 11–30 months (*M* = 20.18, *SD* = 5.18) and their mothers (all from middle-income Chinese families) participated in simulated distress scenarios and structured mother–infant interaction sessions. These paradigms are widely used to study infant empathy and responsive parenting. The maternal empathy levels were measured using the Adult Empathy Scale (E-scale). The results indicate that (1) by their second year, infants largely acquire the capacity for other-oriented empathy and display significantly greater levels of empathy toward their mothers than toward strangers; (2) maternal empathy is significantly and positively correlated with responsive parenting and infant empathy, responsive parenting is significantly and positively correlated with infant empathy; and (3) responsive parenting fully mediates the effect of maternal empathy on infant empathy. These findings indicate that maternal empathy level can be enhanced to improve the quality of parent–child interaction, thereby promoting infant empathy development.

## 1. Introduction

Empathy is an emotional response that stems from an understanding of another person’s emotional state or condition (Decety and Svetlova, 2012). It has two components: emotional and cognitive empathy (Davis, 1983; Zahn-Waxler et al., 1992; Eisenberg, 2000). Emotional empathy is the ability to respond to others’ the emotional feelings, whereas cognitive empathy is the ability to perceive, cognize, and understand these feelings. Young children exhibit both forms of empathy from an early age. For instance, some studies have reported young children’s inquiry behaviors, which is a cognitive form of hypothesis testing (Gladstein, 1983; Landry et al., 2006). Other studies have documented young children being affected by the painful cries of their peers, paying attention to others’ pain, and experiencing and expressing concern for others (Simner, 1971; Roth-Hanania et al., 2011; Davidov et al., 2013; Liddle et al., 2015; Abramson et al., 2019; Davidov et al., 2020). Empathy helps promote responsive and caring behaviors for others’ welfare and has meaningful links to prosocial behaviors such as helping, sharing, comforting, and cooperating (Hoffman, 1975; Simmons, 2014).

During early childhood, empathy development relies on the progressive maturation of brain circuits and neural representations, constructed through interaction with the social environment (Dahl and Freda, 2017; Decety and Holvoet, 2021). Among family factors, scholars believe that parental dispositions (such as empathy), especially those of the mother (Fabes et al., 1990; Farrant et al., 2012), are related to infants’ empathy development (Barnett et al., 1980; Eisenberg et al., 1991; Guo and Feng, 2017; Ng et al., 2020; Salvadori et al., 2021). Thus, excluding the influence of heredity, understanding the mechanisms through which parents’ empathy influences children’s empathy development would help provide caregivers with a clear practical framework and aid scholars in tracing the origins of individual differences in empathy during early childhood.

Early scholars have suggested that the development of infant empathy is stage specific. They undergo developmental processes, such as empathic arousal and other**-**oriented empathy from the first to the second year of their lives (Hoffman, 1975; Zahn-Waxler et al., 1979, 1992; Raboteg-Saric and Hoffman, 2001; Knafo et al., 2008). However, later scholars disagreed with this stage-specific explanation and showed that the emotional and cognitive dimensions of empathy emerge in the first year of life (Davidov et al., 2013; Uzefovsky et al., 2019), and gradually increase in the second year (Roth-Hanania et al., 2011). Upshaw et al. (2015) studied individual differences in empathy of infants aged 12–15 months based on their pupillary changes; they confirmed that infants could show high arousal in response to others’ emotional displays by the end of their first year. That is, individuals begin to exhibit some relatively mature empathic responses from the end of the first year of their lives to the beginning of the second year. Therefore, to further explore these findings, it is worth measuring infants’ empathetic responses before and after the second year of their lives.

Previous studies measuring the level of infant empathy have mostly been unidimensional and lack a more generalized perspective to explain empathy development across infancy. For instance, although previous studies have explored different aspects of infant empathy, they have focused more on the co-variation between dimensions (Davidov et al., 2013; Uzefovsky et al., 2019) or have focused on a single dimension of infant empathy development (Davidov and Grusec, 2006; Brownell, 2016; Davidov et al., 2020), without exploring the developmental differences between the dimensions. Moreover, in previous studies, the initial response of infant empathy was observed in the context of crying peers (Simner, 1971; Liddle et al., 2015). Infants showed stronger empathy toward their mothers than toward other unknown adults (Zahn-Waxler et al., 1979, 1992; Kochanska, 1998; Kiang et al., 2004; Moreno et al., 2008; Roth-Hanania et al., 2011). This finding indicates that infant empathic development for this kind of empathy is limited to specific objects (such as peers and mothers). Discussions of infant empathy typically revolve around infants below the age of two (Kochanska, 1998; Kiang et al., 2004; Moreno et al., 2008; Roth-Hanania et al., 2011). The question that arises is whether infants will empathize with more objects as they grow up. A study has shown that the development of infant empathy is common across mothers and strangers as they get older (Knafo et al., 2008). Therefore, investigating the developmental sequence of empathy’s internal structures during the second and third years of infants’ lives would broaden previous studies and help explain the differences in infants’ empathy toward their mothers and strangers.

As mentioned earlier, parental traits (e.g., empathy) are considered important influences on child empathy development (Barnett et al., 1980; Eisenberg et al., 1989, 1991; Ng et al., 2020). Some twin studies have found that individual differences in infant empathy result from a confluence of genetic and environmental factors, which contain common elements related to parental empathy (especially maternal empathy; Volbrecht et al., 2007; Knafo et al., 2008). Infants are extremely sensitive to emotional signals from caregivers (e.g., mothers), and their physiological connection involves an “emotional contagion” between mother and child (Feldman, 2016). Thus, maternal empathy can be hypothesized to have a strong relationship with the development of infant empathy. For example, Upshaw et al. (2015) study, in which higher pupil dilation in infants’ responses to others’ emotional displays was connected with maternal empathy. However, thus far, most studies that have examined the connection between mothers’ and their children’s empathy merely performed a correlational analysis (Barnett, 1987; Fabes et al., 1990; Eisenberg et al., 1991; Farrant et al., 2012). Only a few exceptional studies have been discussed more intensively. Meidan and Uzefovsky (2020) discovered that the association between maternal cognitive empathy and child’s cognitive empathy is mediated by the mother’s perceptions and attitudes. Moreover, Strayer and Roberts (2004) discovered that children’s anger mediated the influence of parental empathy on children’s empathy. Therefore, many issues regarding the relationship between maternal and infant empathy require further research to obtain more definitive answers. For instance, what are the pathways through which maternal empathy influences the development of children’s empathy? Are the pathways related to parenting behaviors that are considered very important?

Parenting style is strongly associated with individual empathy development (Hoffman, 1975; Barnett, 1987; De Haan and Gunnar, 2009; Grusec, 2011; Guo and Feng, 2017). Children of parents who are warm, supportive, sensitive, and low in negativity tend to show higher levels of empathy (Zhou et al., 2002; Laible et al., 2004; Schuhmacher et al., 2017). Researchers have used mainly “responsiveness levels” to measure the quality of early parenting (Hall and Copeland, 1972), and have found that responsive parenting predicts and facilitates children’s empathy development (Eisenberg et al., 1992; Davidov and Grusec, 2006; Malti et al., 2013; Daniel et al., 2016). According to the attachment theory explanation, early emotional experiences between infants and their caregivers are essential for the emergence and development of empathy in infants. Panfile and Laible (2012) pointed out that children who were securely attached to their caregivers, and therefore felt safe and loved, were subsequently more sensitive to the emotional needs of others. Thus, responsive parenting enables children to feel safe and develop basic trust in their caregivers and environment, leading to a more secure attachment with their parents (Eisenberg et al., 1992; Leerkes, 2010; Waters et al., 2010; Ciciola et al., 2013; Leerkes et al., 2015; Stern and Cassidy, 2017). On the contrary, if the communication between an infant and caregiver breaks down and is characterized by inconsistent and unresponsive interactions, the relationship may lack trust and security, hindering the infant’s subsequent social and emotional development (Roth-Hanania et al., 2011).

Any parenting style used while interacting with an infant is bound be influenced by the nurturer’s characteristics (Hu et al., 2020; Ojo et al., 2021). Research has shown that mothers with high empathy levels are more capable of creating an intimate, multi-responsive atmosphere during infant care. In particular, mothers with high levels of perspective taking use more responsive parenting (Kochanska, 1998; Kochanska et al., 2004). Higher maternal empathy levels predict sensitive and reactive parenting, and lower levels of maternal empathy predict harsh and intrusive parenting (Trentacosta and Shaw, 2007; Emery et al., 2014; Leerkes et al., 2015; Krauthamer Ewing et al., 2019). Dix (1992) argued that empathy is central to a sensitive, responsive parenting approach because empathic responses facilitate a child-centered approach to parenting and are in harmony with the child’s emotional state, interests, and needs. Mothers’ ability to empathize helps them feel and interpret their children’s emotions acutely and accurately, and in turn, act on their children’s needs and adapt caregiving behavior (Batson et al., 1987; Eisenberg et al., 1992; George and Solomon, 2008). Accordingly, they are more capable of establishing intimate and mutually responsive interactions with their child from an early age (Kochanska, 1997; Decety and Holvoet, 2021). Thus, responsive parenting may help explain the relationship between maternal and infant empathy.

Based on the aforementioned background, this study contributes to existing research by investigating (1) the development of infant empathy and the difference with across-object empathy; (2) the relationship between maternal empathy, responsive parenting, and infant empathy; and (3) whether responsive parenting mediates the relationship between maternal and infant empathy.

The study involves situational observation of infants aged 11–30 months (i.e., before and after their second year) and their mothers’ conducted in the Chinese cultural context. Meanwhile, the E-scale was used to measure the maternal empathy. We observed structured mother–infant interaction activities using non-participatory observation and graded responsive parenting using Kochanska et al. (2004) coding rules. Furthermore, we adopted the simulated distress scenarios and its coding rules designed by Zahn-Waxler et al. (1992) to record and code infants’ empathic responses.

## 2. Materials and methods

### 2.1. Participants

We recruited 35 neurotypical infants (17 girls) and their mothers, all of whom came from middle-income families in Jilin, China, and completed a 20-day data collection through a public recruitment process. Two infants were excluded because of their maladaptive behavior in mother–infant interaction. Thus, the final sample size consisted of 15 girls and 18 boys (*N* = 33, age range = 11**–**30 months, *M* = 20.18, *SD* = 5.18). The number of participants was limited to gain accurate experimental records in a limited development period (Negayama et al., 2015). Infants and mothers provided data on mother–infant interaction, stranger–infant interaction, and adult empathy. Most mothers (*N* = 33, *M* = 31.42) had a bachelor’s degree or above (only two mothers had a junior high school degree). Before collecting data, we explained the purpose of this study to the mothers to obtain their informed consent. The participants’ details were anonymized to protect their privacy.

### 2.2. Procedures and measures

Data were collected in the participants’ homes to capture the infants’ real reactions in a familiar and quiet environment. Two female researchers visited the participants’ homes. First, the mothers filled in the basic information and E-scale, after which we measured infant empathy and responsive parenting through a non-participatory situational observation. Both infants and their mothers participated in the situational observation, and the whole process was videotaped. Observations were usually arranged when infants felt their best, such as after a nap or a meal. If not, the researchers rescheduled the visit. Each visit was completed within 1.5 h, including 5 min for the mother to fill in the scale, and 10**–**20 min for the researchers to talk and interact with the infant and the mother (so that the infant can get familiar with the researchers and the presence of the camera), 20–30 min for observing the situation of simulated distress, and 30 min for observing the mother**–**infant interaction. The coders of this study were all graduate students majoring in developmental psychology and had undergone strict training.

#### 2.2.1. Maternal empathy measurement

Maternal empathy was measured using the E-scale compiled by Leibetseder et al. (2007). The E-scale consists of 25 topics covered by four subscales: cognitive sensitivity, emotional sensitivity, cognitive concern, and empathy concern. These subscales can be combined into two dimensions: emotional empathy (emotional sensitivity and empathy concern) and cognitive empathy (cognitive sensitivity and cognitive concern) (Tran et al., 2013). The participants rated their responses on a 5-point Likert scale, where (0 = *totally inconsistent*, 1 = *slightly consistent*, 2 = *slightly consistent*, 3 = *relatively consistent*, and 4 = *very consistent*).

After a language translation, the Chinese version of E-scale was tested and applied in the Chinese cultural context for the first time. Thus, the scale was first revised and then used as a formal measure of maternal empathy. The scale was distributed randomly on the Internet twice. Overall, 258 completed questionnaires were received for the first time, of which 248 were effective (efficiency rate = 96.12%). The average age of the participants was *M* = 27.05 years, and the standard deviation was *SD* = 6.09. After assessing the first batch of data using SPSS27.0 and AMOS26.0, the entries with insignificant loadings in the emotional empathy dimension (Q1, Q11, and Q20) were removed. The second time, 256 questionnaire copies were collected, of which 248 were valid (efficiency rate = 96.88%). The average age of the participants was *M* = 24.33 years, and the standard deviation was *SD* = 4.70. Confirmatory factor analysis (CFA), CFI = 0.981, GFI = 0.963, and TLI = 0.972 were all greater than 0.90; *χ^2^*/*df* = 1.988 < 3, RMSEA = 0.063 < 0.08. Therefore, the revised scale was suitable for use in the current research.

#### 2.2.2. Infant empathy measurement

The measurement paradigm of infant empathy was based on the research paradigm used by Zahn-Waxler et al. (1992) in their study of infant empathy: creating a simulated distress for a mother, companion, or stranger; observing and recording the infant response to the sadness of the mother, companion or stranger; and coding the video. In this study, we investigated the infants’ reaction to the sadness and pain experienced their mother and a stranger by creating the following situation.

##### 2.2.2.1. Situation creation and observation

Step 1: Introduce the experimental instructions. First, researcher B familiarized the infant with the camera. Meanwhile, researcher A clearly explained the instructions concerning experimental observation to the mother. The instructions were as follows: “This experimental study aims to observe the daily activities of the infant, not the mother, so you can interact with your baby as usual.

We will not participate directly during the observation. If we appear at the experimental site, please do not look at us or talk to us and do not guide the infant to look at or communicate with us either. You should try to ignore us. After about 10 min, I will ask you to pretend to hit the wall or the corner of the table, and to feel pain for 60 s (simulated distress). I will inform you when the time is up, but in the process, do not call your baby or look at him/her.” When mother understood the instructions, researcher B turned on the video camera.

Step 2: Create separate situations in which the mother and stranger would pretend to be distressed and observe infant empathy in both situations. First, the mother and infant were allowed to move freely, and the mother was free to take the infant to do activities with which they were familiar. At the end of the free activity (i.e., after 10 min), the mother pretended to be injured for 60 s (note that the mother was not supposed to look at the infant at this time to avoid the appearance of a specific request**–**response). After the researcher gave the signal, the mother stopped pretending and continued the game. After a few minutes, the mother told the infant, “××, mommy is going to the bathroom, let auntie (pointing to researcher A) play with you for a while, OK?” After obtaining the infant’s consent, she left.

After the mother left, the researcher asked the infant, “Shall I play with you for a while?” and then joined the game. After about 5 min, the researcher pretended to be hit by the table foot or arm and pretended to be hurt for 60 s (again, the researcher did not look at the infant and avoided a specific request reaction). Then, the researcher stopped pretending to be in pain when she received the signal to stop. Finally, the researcher took out a gift box and told the mother and infant to open it together. After 2 min, the observation ended.

##### 2.2.2.2. Coding

The infants’ behaviors during simulated distress were recorded and coded by referring to and synthesizing the coding rules adopted in Zahn-Waxler et al. (1992) and related studies (Knafo et al., 2008; Roth-Hanania et al., 2011). The infants’ performance was coded from three aspects, namely empathic concern (emotional empathy index), hypothesis testing (cognitive empathy index), and prosocial behavior (prosocial behavior index). The coding was performed by two coders through consultation. We coded infants’ performance during the 60 s in which the mother or researcher pretended to be in pain until she received the signal to stop. The score range of each dimension was 1–4. Cronbach’s alpha encoded by the two researchers was 0.81, 0.86, and 0.86, respectively, for each dimension.

###### 2.2.2.2.1. Empathic concern

The infants’ obvious emotional expression, including facial, verbal, gesture, and body posture, in response to a person’s (their mother’s or stranger’s) distress. Infants were scored on a 4-point scale, 1 = *none*; 2 = *low* (expression of concern, such as facial expression: frown, but for a relatively short time); 3 = *moderately* (a relatively long expression of care, usually 3–5 s); and 4 = *high* (persistent sympathetic, sad expression or sympathetic tone).

###### 2.2.2.2.2. Hypothesis testing

Inquiry behavior in terms of language and body movements indicates that the infant is trying to detect pain or cognitively understand what happened to the injured person. Infants were rated on a 4-point scale: 1 = *none*; 2 = *simple nonverbal* (touching the body part of the injured person or looking at the injured person’s face) or *simple verbal inquiry*; 3 = *combination of verbal and nonverbal inquiry* (single combination); and 4 = *repeated or relatively complicated attempts to understand the pain of others*.

###### 2.2.2.2.3. Prosocial behavior

Trying to help or comfort the injured. Infants were rated on a 4-point scale: 1 = *none*; 2 = *light help* (such as a pat); 3 = *moderate help* (helping behavior lasts for 3–5 s, or repeated prosocial behavior); and 4 = *long-term help* (over 5 s).

#### 2.2.3. Responsive parenting measurement

Responsive parenting was recorded, observed, and analyzed during unstructured regular activities between mothers and infants in which they interacted in their natural and daily states. The parenting styles of the mothers were coded using reactivity level as an index.

##### 2.2.3.1. Situation creation and observation

Observation of a structured parent–child interaction required the mother to interact with the infant for 30 min, which includes regular activities (10 min) and interactive activities and games with toys (20 min). The game was about fruit cutting, and toys were provided by the researchers. Finally, the infant and mother opened a gift together (video: 2 min).

##### 2.2.3.2. Coding

Responsive parenting was coded according to Kochanska et al. (2004, 2013) criteria. The level of early parent–child interaction was evaluated on a scale of 1–5 in four areas: coordination routines, harmonious communication, mutual cooperation, and emotional ambiance. High mutual responsiveness levels are defined as the caregiver (generally the mother) giving warm, supportive, sensitive, and consistent care to the child in the areas of exploratory behavior, goal orientation, cognitive performance, curiosity, and problem-solving. During the coding process, both coders together watched the entire contextual video carefully and discussed it.

The coders focused on the parent and child as a pair rather than as individuals and observed their interaction during three time periods (consisting of unstructured routine time and two 10-min intervals of free activities with toys). Cronbach’s alpha encoded by the two researchers was 0.97. The parenting style for each pair is described below, on a 5-point Likert scale ranging from 1 (unresponsive) to 5 (most responsive).

1. Unresponsive: Very low mutual reactions and a bad relationship as they showed some or all of the following behaviors: antagonism, detachment, unresponsiveness, hostility, and emotional negativity. The following behaviors were rarely observed: mutual reaction, coordination, harmony, synchronization, mutual adaptation, cooperation, and emotional positivity.
2. Marginally responsive: A low-level of mutual reaction and not a very good relationship indicated by one or more of the following behaviors: antagonism, detachment, unresponsiveness, hostility, and emotional negativity. The following behaviors were rarely observed: mutual reaction, coordination, harmony, synchronization, mutual adaptation, cooperation, and emotional positivity.
3. Moderately responsive: Parent–child interaction fluctuates between low and high level of mutual reactions and responsiveness or is at an average level (neither high nor low).
4. Highly responsive: Reasonable level of mutual reaction and a cordial relationship indicated by one or more of the following behaviors: mutual reaction, coordination, harmony, synchronization, mutual adaptation, cooperation, and emotional positivity. The following behaviors were rarely observed: confrontation, detachment, unresponsiveness, hostility, and emotional negativity.
5. Most responsive: Very high level of mutual reaction and a good relationship characterized by a strong and consistent presence of the following behaviors: mutual reaction, coordination, harmony, synchronization, mutual adaptation, cooperation, and emotional positivity. The following behaviors were rarely observed: confrontation, detachment, unresponsiveness, hostility, and emotional negativity.

For the rating, the coders needed to comprehensively consider the following dimensions and definitions:

###### 2.2.3.2.1. Coordinated routines

Low: No routine is established between parents or children, who share an unstable relationship with inconsistent expectations, leading to conflicts. High: There is a relaxed, comfortable, and coordinated routine between parents and children. High expectations regarding common routines are evident and a close relationship is promoted.

###### 2.2.3.2.2. Harmonious communication

Low: There is little or no communication between parents and children. High: Continuous and fluent verbal and nonverbal communication involving high reciprocity.

###### 2.2.3.2.3. Mutual cooperation

Low: Parents and children cannot accept their roles (frequent autonomy struggle and/or resistance), and conflicts escalate and get out of control. High: Parents and children can effectively solve potential conflicts. The mother and child adopt an acceptable and willing attitude toward each other’s influence and are in psychological harmony with one another.

###### 2.2.3.2.4. Emotional ambiance

Low: Parents and children experience obvious negative emotions. A negative atmosphere pervades the interaction, and positive emotions are nonexistent. High: Parents and children enjoy a positive emotional atmosphere, which indicates that they are very happy in each other’s company, characterized by warmth and joy. Parents and children can effectively deal with the occurrence and negative effects of painful events. There is a natural outpouring of emotions in the interaction, and for both the parent and the child, expressing one’s emotions is a source of happiness.

## 3. Results

### 3.1. General situation of empathy in infancy

Table 1 shows the average and standard deviation of empathic concern, hypothesis testing, and prosocial behavior of infants when mothers and strangers simulate distress. If other-oriented empathy occurs in infancy, the average should be around 2 (slightly). As can be seen from Table 1, the average scores of infants’ empathic concern, hypothesis testing, and prosocial behavior toward their mothers were in the range of 2 or higher (2–4). In addition to prosocial behavior, the average empathy level for strangers was greater than 2. This reflects the emergence of other-oriented empathy during infancy.

**Table 1:** Descriptive and ANOVA analysis of infant empathy development (*N* = 33).

	Empathic subdimension	Empathic object *M* (*SD*)		*F*	*df*	*η* ^2^_p_
		Mother	Stranger			
Empathic subdimension score	EC	3.24 (0.90)	3.06 (1.12)	33.18***	2	0.34
	HT	2.76 (0.83)	2.39 (0.75)			
	PB	2.73 (1.04)	1.70 (1.05)			
Empathic object				7.39**	1	0.10
Empathic subdimension × empathic object				7.38**	2	0.10

EC, empathic concern; HT, hypothesis testing; PB, prosocial behavior. **p < 0.01; ***p < 0.001.

According to the preliminary statistical test results, infants’ empathy scores were not affected by their sex and age (in months) for both maternal and stranger empathic tasks (*p* > 0.05). Therefore, sex and age were not considered for subsequent statistical analysis.

Analysis of variance (ANOVA) was repeatedly measured for the infant empathic subdimensions (empathic concern, hypothesis testing, and prosocial behavior) and the empathic objects (mothers and strangers). The results are presented in Table 1. The main effect of the infant empathic subdimensions was statistically significant [*F*(2, 60) = 33.18, *p* < 0.001, *η*^2^_p_ = 0.34]. A *post-hoc* test showed that the mean difference between the groups was statistically significant. Moreover, the score of infant empathic concern was higher than that for hypothesis testing and prosocial behavior (*M*EC-HT = 0.58, *p* < 0.001; *M*EC-PB = 0.94, *p* < 0.001), and the score of hypothesis testing was higher than that for prosocial behavior (*M*HT-PB = 0.36, *p* < 0.01).

The main effect of empathic objects was significant [*F*(1, 60) = 7.39, *p* < 0.01, *η*^2^_p_ = 0.10], and the score of empathy for mothers was higher than that for strangers (*M* = 0.53, *p* < 0.01).

The subdimensions of infant empathy and empathic objects had a significant interaction effect [*F*(2, 60) = 10.88, *p* < 0.01, *η*^2^_p_ = 0.15]. The results of the simple effect analysis showed that when the empathic object was the mother, the effect of the empathic subdimension was significant [*F*(2, 63) = 5.11, *p* < 0.01, *η*^2^_p_ = 0.14], and the score for empathic concern was higher than that for hypothesis testing and prosocial behavior (*M*EC-HT = 0.49, *p* < 0.01; *M*EC-PB = 0.52, *p* < 0.01). When the object of empathy was a stranger, the effect of the empathic subdimension was significant [*F*(2, 63) = 28.04, *p* < 0.001, *η*^2^_p_ = 0.47], and the score for empathic concern was higher than that for hypothesis testing and prosocial behavior (*M*EC-HT = 0.68, *p* < 0.001; *M*EC-PB = 1.36, *p* < 0.001); the score for hypothesis testing was higher than that for prosocial behavior (*M*HT-PB = 0.70, *p* < 0.001). At the subdimension level, the score for prosocial behavior of infants was significantly different [*F*(2, 60) = 16.13, *p* < 0.001, *η*^2^_p_ = 0.20], and the score for infants’ prosocial behavior toward their mothers was significantly higher than that for strangers.

### 3.2. Correlation analysis between maternal empathy, responsive parenting, and infant empathy

As the current study focuses more on the relationship between maternal and infant empathy, the empathic response to strangers was not analyzed. Descriptive statistics and correlation analysis of each variable are shown in Table 2. The results showed that maternal cognitive empathy, emotional empathy, and the total empathy score were positively and significantly correlated with infant empathic concern, hypothesis testing, and the total empathy score. No significant correlation was found with prosocial behavior. Responsive parenting was positively correlated with infant empathy, hypothesis testing, prosocial behavior, and the total empathy score. Thus, maternal empathy and responsive parenting were closely related to infant empathy. Maternal cognitive empathy, emotional empathy, and the total empathy score were significantly positively correlated with responsive parenting. Thus, the higher the maternal empathic capacity, the more inclined the mothers were to adopt responsive parenting.

**Table 2:** Descriptive and correlation analysis of study variables (*N* = 33).

	*M*	*SD*	1	2	3	4	5	6	7	8
Maternal empathy										
1. Cognitive empathy	12.74	2.51	1.00							
2. Emotional empathy	13.10	2.80	0.76***	1.00						
3. Total score	25.84	4.98	0.93***	0.95***	1.00					
4. Responsive parenting	11.70	3.04	0.57**	0.49**	0.56**	1.00				
Infant empathy										
1. Empathic concern	3.24	0.90	0.37*	0.40*	0.41*	0.70***	1.00			
2. Hypothesis testing	2.76	0.83	0.52**	0.40*	0.49**	0.71***	0.62***	1.00		
3. Prosocial behavior	2.73	1.04	0.24	0.28	0.28	0.51**	0.54**	0.61***	1.00	
4. Total score	8.73	2.36	0.43**	0.42*	0.45**	0.74***	0.84***	0.86***	0.86***	1.00

*p < 0.05; **p < 0.01; ***p < 0.001.

### 3.3. Mediating role of responsive parenting in maternal and infant empathy

**Figure 1:**
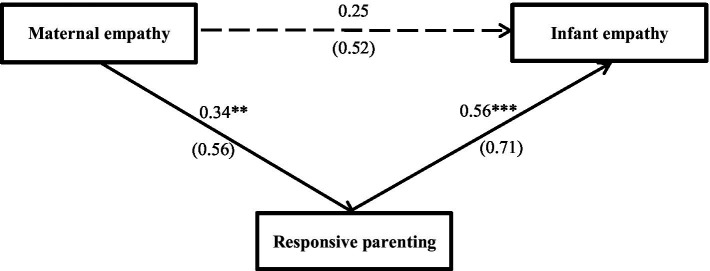
The relationship between maternal and infant empathy as mediated by responsive parenting. The solid line in the figure in that the path coefficient is significant, and the dotted line indicates that the path coefficient is not significant. Unstandardized path coefficients (standardized coefficients in parentheses). **p < 0.01; ***p < 0.001.

This study used the PROCESS plug-in of SPSS 27.0 to analyze and evaluate the mediating effect of responsive parenting. The indirect effect was significant when evaluating the mediating effect and estimating the confidence interval by deviation-corrected nonparametric percentage bootstrap (repeated sampling 1,000 times) and when the 95% confidence interval did not contain 0, it indicated that the indirect effect was significant. The results showed that the mediating effect of responsive parenting between maternal and infant empathy was 0.19. The 95% confidence interval was (0.10, 0.30), and the mediation effect was significant. Moreover, after controlling for the mediating variable responsive parenting, the independent variable, maternal empathy, had no significant influence on the dependent variable, infant empathy. Furthermore, the confidence interval was (−0.12, 0.17), which contains 0. Therefore, responsive parenting mediated maternal empathy’s influence on infant empathy (Figure 1).

## 4. Discussion

### 4.1. Development of infant empathy

This study revealed that most infants already have a stable initial empathy as they are aware of others’ pain when an adult (mother or stranger) gets injured and try to comfort and help the injured person using their language, gestures, and other actions. However, this study also found that there are differences in the developmental sequence of the three subdimensions of empathy. The survey results suggest that among the three subdimensions, infant empathy scored the highest, followed by hypothesis testing and prosocial behavior. This result confirms prior results to some extent. Prior studies have shown that 3-month-old infants have basic empathy toward other’s suffering, as shown in their facial expressions, voices, and gestures. From 3 to 36 months, children’s empathy gets more complicated and increasingly accompanies communication and prosocial behavior (efforts to help or comfort others) (Davidov et al., 2020). The differences in empathy development may be owing to the traits of each subdimension and the patterns of early development. Empathic concern is part of emotional empathy, which is similar to emotional contagion and emotional resonance. Emotional contagion is a diffusion of emotional stress and does not require conscious effort (Decety and Cowell, 2014). Therefore, empathic concern appears earlier in infancy compared to other dimensions (Hay et al., 1981; Liddle et al., 2015). In contrast, the development of prosocial behavior depends more on daily communication (Brownell, 2016), which requires more cognitive resources (Decety and Meyer, 2008); thus, infants may need more time to develop prosocial behavior.

The present study also analyzed infant empathy performance for different adult objects. The findings showed that infant empathy score for their mother was significantly higher than that for strangers, which was consistent with previous research findings (Zahn-Waxler et al., 1979, 1992; Kochanska, 1998; Kiang et al., 2004; Moreno et al., 2008; Roth-Hanania et al., 2011). The findings also showed that infants’ empathy score for their mother was significantly higher than for strangers on the prosocial dimension, which was not the case for empathic concern and hypothesis testing. Infants were more likely to exhibit prosocial behavior toward their mothers than strangers, implying that infant empathy development has not yet demonstrated cross-object stability, which echoes the findings on later development of prosocial behavior in infant empathy. However, these results could also be attributed to the fact that infants have more life experiences with their mothers. For example, infants have had opportunities to console and help their mothers in the past, and they have gained more relevant experiences regarding how to respond prosocially to their mothers (Scrimgeour et al., 2013). Concurrently, mothers give social rewards to their infants (e.g., smiles, praise, affection) and encourage them to repeat this prosocial response in other situations (Dahl, 2015; Davidov et al., 2020). In short, the current findings showed that infant empathy develops in diverse ways when they encounter various adults, which enrich previous research and findings.

### 4.2. Relationship between maternal empathy, responsive parenting, and infant empathy

#### 4.2.1. Relationship between maternal and infant empathy

The present study found that maternal empathy was partially correlated with infant empathy. The results showed that the total empathy score and the subdimensions of maternal empathy (emotional empathy and cognitive empathy) were significantly correlated with the total score and subdimensions of infant empathy (empathic concern and hypothesis testing), respectively, which was consistent with the results of two recent studies and fully illustrates the strong association between maternal and infant empathy. Salvadori et al. (2021) showed that parents who scored higher on emotional empathy tended to have infants who showed more empathy-related behavior, such as emotional mimicry. Upshaw et al. (2015) also showed that the early variability of empathy in infancy is closely related to the empathy tendency of parents. These results indicate that there was a certain correlation between individual differences in infant empathy development and maternal empathy level. Furthermore, this study also found that maternal empathy was not significantly correlated with infants’ prosocial behavior. One possibility for this result is the inter-dimensional correspondence and developmental maturity (Roth-Hanania et al., 2011; Davidov et al., 2020). That is, prosocial behaviors demand higher level of self-regulation, with the emotion, cognition, and action, to facilitate a more complex integration of that coaction. (Zahn-Waxler et al., 1992; Decety and Meyer, 2008; Davidov et al., 2013).

#### 4.2.2. Relationship between responsive parenting and infant empathy

This study found that responsive parenting was significantly related to infant empathy, which was consistent with some of the previous research results (Eisenberg et al., 1992; Davidov and Grusec, 2006; Malti et al., 2013; Daniel et al., 2016). Responsive parenting is key to infant development, and allows infants to become more emotionally stable, social, and empathic while showing greater concern for caregivers. Thus, responsive parenting predicts and promotes infants’ empathy development (Eisenberg et al., 1998). Koestner et al. (1990) 26-year follow-up study on the family origins of empathic concern showed that some dimensions of parenting style were significantly highly correlated with children’s later empathic concern abilities. These findings all point to the importance of responsive parenting for infants’ cognitive and emotional development.

#### 4.2.3. Relationship between maternal empathy and responsive parenting

The present study found that maternal cognitive empathy, emotional empathy, and total empathy scores were significantly and positively correlated with responsive parenting, indicating that mothers with higher empathy tend to adopt responsive parenting. This finding was consistent with previous research findings (Kochanska, 1998; Kochanska et al., 2004; Krauthamer Ewing et al., 2019; Decety and Holvoet, 2021). Related studies have shown that mothers with high empathy were more capable of establishing a positive emotional ambiance during infant care (Kochanska, 1998; Kochanska et al., 2004), responsive to infant emotional stimuli (Kalliopuska, 1984; Davidov et al., 2020), and capable of establishing an intimate and responsive relationship with the child early in life (Decety and Holvoet, 2021). Therefore, we can speculate that maternal empathy is a key factor in determining the responsive parenting level in day-to-day mother–infant interactions. Moreover, infancy is not only a critical period for the development of parent–child relationship (Daniel et al., 2016), but also the best time to promote responsive parenting (Landry et al., 2006). Therefore, responsive parenting during parent–child interactions is crucial for infant development.

### 4.3. Mediating role of responsive parenting in maternal empathy and infant empathy

The results of the mediation analysis showed that maternal empathy was not a significant predictor of infant empathy, and that responsive parenting fully mediated the relationship between maternal and infant empathy. Few studies have investigated this topic except for one, which involved groups of young children, school-age children, and adolescents (Strayer and Roberts, 2004). In fact, there is a close correlation between maternal empathy, responsive parenting, and infant empathy. Mothers who have a high level of maternal empathy are more likely to adopt a warm, sensitive, responsive parenting and are more capable of creating an intimate and sensitive, multi-responsive atmosphere for their infants (Kochanska, 1997; Kochanska et al., 2004), and are more capable of adapting caregiving behavior to meet their infants’ needs (George and Solomon, 2008). Thus, maternal empathy provides the optimal framework for infants’ social emotional, cognitive, and physical development, especially the development of infant empathy (Gottman et al., 1996; Katz and Gottman, 1997). In summary, the present study found that responsive parenting fully mediates the relationship between maternal and infant empathy.

### 4.4. Research limitations and prospects

This study had some limitations, which are outlined below. We also suggest some directions for future research by pointing out the ways in which these limitations can be addressed.

First, the current research on infant empathy mainly focuses on infants’ empathy in response to others’ emotions of distress (Liddle et al., 2015; Davidov et al., 2020). However, the study did not consider other emotions, for example, other people’s positive emotions (e.g., happiness) or different negative emotions (e.g., anger and disgust). Therefore, future studies could explore a better research paradigm to accurately measure infant empathy in response to other kinds of emotions or investigate whether infants have limited ability to empathize with positive emotions in the first few years of their lives.

Second, this study only analyzed the relationship between maternal empathy and infant empathy from the perspective of parent–child interactions, without introducing or controlling for more variables (e.g., individual infant factors). Thus, more comprehensive study could be conducted in the future by incorporating other variables. Empathy develops gradually throughout early life and is influenced by a range of factors, including genetics, temperament, context, and environment (Decety and Holvoet, 2021). Previous studies have confirmed the role of temperamental traits in infant empathy development (Schuhmacher et al., 2017). For example, Robinson (1994) found that specific attributes of temperament, such as inhibition, fear, and sadness among toddlers, were negatively related to their empathy and comfort behavior. Hammond and Carpendale (2015) found that the sociability of infants in the first year of their lives was positively related to emotional empathy and instrumental help. Thus, the individual differences in infant empathy may be affected by temperament factors and may even affect the relationship between maternal empathy and infant empathy. Therefore, it is necessary to explore more relevant variables in future research.

Finally, most studies have been conducted from the behavioral perspective of infant empathy. Thus, scholars should aim to diversity their methods to investigate empathy performance in early life in the future. Empathy is a complex social cognitive ability whose physiological basis involves multiple brain regions (Decety and Svetlova, 2012). Moreover, empathy development early in life depends on the gradual maturation of brain circuits and neural representations (Decety and Holvoet, 2021). Considering the development of empathy in infants only from behavioral indicators is bound to have limitations, such as researcher stereotypes and confirmation bias, which could lead to erroneous results. Therefore, in the future, infant empathic responses could be measured using functional magnetic resonance imaging, near-infrared spectroscopy techniques, and so on.

## Data availability statement

The original contributions presented in the study are included in the article/supplementary material, further inquiries can be directed to the corresponding author.

## Ethics statement

The study involving human participants were reviewed and approved by the Ethical Committee of Northeast Normal University, China. Written informed consent to participate in this study was provided by the participants and the participants’ legal guardian.

## Author contributions

YZ and ZM collected, coded, and processed the data. HL, XC, and LW drafted the manuscript. HL provided critical revisions. All authors contributed to the article and approved the submitted version.

## Funding

This study was supported by MOE (Ministry of Education in China) Project of Humanities and Social Sciences (grant no. 19YJC190012) and the Research Program Funds of the Collaborative Innovation Center of Assessment toward Basic Education Quality at Beijing Normal University (2021-03-010-BZPK01).

## Conflict of interest

The authors declare that the research was conducted in the absence of any commercial or financial relationships that could be construed as a potential conflict of interest.

## Publisher’s note

All claims expressed in this article are solely those of the authors and do not necessarily represent those of their affiliated organizations, or those of the publisher, the editors and the reviewers. Any product that may be evaluated in this article, or claim that may be made by its manufacturer, is not guaranteed or endorsed by the publisher.
